# Cross-cultural adaptation and psychometric evaluation of the Yoruba version of the Back beliefs questionnaire among patients with chronic low-back pain

**DOI:** 10.1186/s12955-020-01322-2

**Published:** 2020-03-17

**Authors:** C. E. Mbada, O. A. Adeniyi, O. A. Idowu, C. T. Fatoye, A. C. Odole, F. Fatoye

**Affiliations:** 1 Department of Medical Rehabilitation, College of Health Sciences, Obafemi Awolowo University, Ile – Ife, Nigeria; 2 Department of Physiotherapy, School of Basic Medical Sciences, College of Medical Sciences, University of Benin, Benin City, Nigeria; 3 Department of Health Professions, Faculty of Health, Psychology and Social Care, Manchester Metropolitan University, Manchester, UK; 4 Department of Physiotherapy, Faculty of Clinical Sciences, College of Medicine, University of Ibadan, Ibadan, Nigeria

**Keywords:** BBQ, Low back pain, Psychometric evaluation, Translation

## Abstract

**Purpose:**

To translate, culturally adapt and conduct a psychometric evaluation of the Yoruba version of the Back Beliefs Questionnaire (BBQ) among patients with chronic low-back pain.

**Methods:**

The English version of the BBQ was translated into the Yoruba language through a process of forward-backward translation, reconciliation and harmonization of the reconciled items sequentially. Thereafter, Cronbach’s Alpha, Intra-Class Correlation (ICC), Bland-Altman’s analysis were used to determine the internal consistency, test-retest reliability and limits of agreement of the Yoruba version of the BBQ (BBQ-Y). Other psychometric properties of the BBQ-Y explored comprised acceptability, standard error of measurement (SEM), minimal detectable change (MDC), convergent validity and floor and ceiling effects. While 119 respondents participated in the validity testing, only 51 of them were involved in the reliability testing of the BBQ-Y. Data were analysed using descriptive and inferential statistics. Alpha level was set at *p* < 0.05.

**Results:**

The mean age of the respondents all the respondents was 56.8 ± 8.5 years. The BBQ-Y had excellent acceptability with a global Cronbach Alpha score of 0.71. The SEM and MDC of the BBQ-Y were 2.3 and 6.4. The BBQ-Y ICC score for test-retest was 0.89, while the Bland-Altman analysis showing limits of agreements for the test-retest reliability were − 6.84 and 5.70. The convergent validity of the BBQ-Y showed a weak correlation (r = 0.273, *p* = 0.001) with pain intensity using the visual analogue scale.

**Conclusion:**

This is the first study to culturally adapt the BBY-Y and determined its psychometric properties. The BBQ-Y has adequate psychometric properties and it is an appropriate outcome measure for use among Yoruba speaking patients with chronic low-back pain.

## Introduction

Low-back-pain (LBP) is among the most costly health care disorders with daily rising costs [1]. Costs associated with LBP comprise direct medical (health-care), indirect (work absenteeism or productivity loss), and direct nonmedical (transportation to meet up with hospital appointments, visits to complementary and alternative practitioners) costs [2]. As such, LBP has become an economic and social burden worldwide and a frequent reason for hospital visits [3]. Further, LBP ranks among the top ten conditions responsible for the highest rates of disability-adjusted life-years worldwide [4]. For most cases of LBP, the patho-anatomical diagnosis is evasive [5]. Consequently, there is increasing consensus that LBP should be viewed from a bio-psychosocial perspective [6, 7].

Biological and psychosocial factors play important roles in the development and chronicity of pain and disability [8]. Specifically, an individual’s belief system about pain is critical to pain chronicity and outcomes of interventions [9]. Irrational beliefs about LBP, is often considered as a signal of an imminent threat, which can cause kinesiophobia, (re) injury, activity limitation, and subsequently persistent disability [9]. Patients with non-specific LBP often believe that certain negative consequences of the pain are unavoidable; and that work, activity, and exercise may cause more pain [10, 11]. This belief that normal activities of daily living are likely to cause excruciating back pain facilitates avoidance or reluctance to engage in activities. Consequently, fear-avoidance behavior is formed, which in turn may cause avoidance of movement that leads to disability [11]. Such negative beliefs represent risk factors for the transition of acute to chronic or long-term LBP. Based on the foregoing, the bio-psychosocial model has been recommended as the best model to understanding LBP [12]. This model recognizes the important interplay between the biological and psychosocial aspects of a person’s pain experience [8]. Psychosocial issues that are related to the perpetuation of LBP include fear of movement, self-efficacy, cultural beliefs and gender [13, 14]. These psychosocial factors may influence a person’s beliefs towards LBP, and possibly contributing to potential disability [14].

Among the designed outcome measures to assess the attitudes and beliefs regarding the future course of LBP is the Back Beliefs Questionnaire (BBQ) [10]. The BBQ examines an individual’s beliefs about back pain and its consequences, regardless of previous experience of back pain. The primary aim of the BBQ is to investigate beliefs about various inevitable aspects of the future owing to low back trouble. The questionnaire has good internal consistency (Cronbach α: 0.7) and test-retest reliability (Intra-Class Correlation (ICC): 0.87) [10]. The BBQ comprises 14 statements to which the respondent shows their level of agreement on a 5 point scale. A score of 1 shows complete disagreement, while a score of 5 shows complete agreement. As five of the 14 (4, 5, 7, 9 and 11) statements are distractors and not usually included in the total scores of the BBQ, the scores of the nine remaining statements (1, 2, 3, 6, 8, 10, 12, 13 and 14) are reversed and then summed to provide a total score ranging from 9 to 45. A lower score shows that the respondent has more negative beliefs about back pain. Because of its psychometric properties and clinical usability, various translations of the BBQ exist. Some of these include the modern standard Arabic [15], Chinese [16], Arabic [17] and French [18] translations.

The need to increase the usage of the BBQ among non-English speakers has resulted in its translation into these various local languages such as the Chinese, French, and modern standard Arabic. Local languages enhance the comprehensibility of scales as cultural groups expression of diseases and use of various health care systems differs [19]. Translating the BBQ into Nigerian languages may, therefore, allow for easy access and understanding of the questionnaire by patients, and it will increase its uptake in the clinical settings. To date, there has been no validated and reliable version of the BBQ in any Nigerian language. Nigeria is the most populous black nation in the world with three main ethnic groups and languages, which are; Hausa, Igbo, Yoruba. According to the Central Intelligence Agency (CIA) world factbook [20], the Yoruba tribe remains one of the largest ethnic groups in sub-Saharan Africa. About 40 million Yoruba people live in Nigeria. In addition, natives from other countries including the Benin Republic, Togo, and Brazil speak the Yoruba dialect. Therefore, the availability of a Yoruba version of BBQ is of importance to the people in this region, as it may help to identify prevalent back beliefs in chronic LBP, as well as inform plan for appropriate intervention. Hence, this study was aimed to culturally adapt the BBQ to the Yoruba language and to determine its internal consistency, test-retest reliability, standard error of agreement, minimal detectable change and floor and ceiling effects among patients with chronic LBP..

## Methods

### Participants and setting

This study design included cultural adaptation, test-retest and cross-sectional psychometric analysis of the Yoruba version of the BBQ. Generally, there is no consensus on sample size on translation and psychometric testing of new tools. However, a sample size of approximately 100 or a minimum of seven participants per variable is deemed sufficient as a general rule for exploratory surveys and for validation of patient-reported outcome tools [21, 22]. Thus for the BBQ with 14 variables, a minimum of 98 participants will be required for validation. A total of 119 respondents chronic LBP defined as having LBP of not less than 3 months consented for the validity testing of the Yoruba translated version of the BBQ, however, only 51 of them took part in the test-retest phase of the psychometric testing. Eligible patients for this study were those who were 18 years or older, literate in Yoruba language, and had no self-reported cognitive impairment were purposively recruited. Individuals with previous history of surgery, psychological disorders, or having red flags suggestive of spinal pathology such as dermatomal sensory loss, myotomic muscle weakness and reduced lower limb reflexes were excluded from the study.

The Health Research and Ethics Committee of the Institute of Public Health, College of Health Sciences Obafemi Awolowo University, Ile-Ife gave approval for this study (IPHOAU/12/778). The study was carried out in three purposively selected hospitals namely the Obafemi Awolowo University Teaching Hospital Complex Ile-Ife, Wesley Guild Hospital, Ilesha and University College Hospital, Ibadan, all in the Southwestern Nigeria. The respondents were recruited from the outpatient physiotherapy departments of the selected hospitals. The respondents gave signed informed consent to participate in the study following full disclosure of the purpose of the study. Two of the researchers (CEM and OAA) inquired about participants’ medical history, screened participants, by asking simple questions to rule out the ‘red flags’ for LBP. This excluded any LBP associated with underlying serious pathology or spinal stenosis. Participants were requested to describe their pain location with a body chart to confirm that pain was in the lower back. One of the authors (OAA) delivered the questionnaires used in this study to eligible respondents by hand. The questionnaires were the Yoruba versions BBQ (BBQ-Y) and the Visual Analogue Scale (VAS-Y) on the day of enrolment (T1). Respondents who consented to participate in the retest phase of the study filled the BBQ-Y after seven days (T2).. In addition, socio-demographic information, as well as data on weight, height, and Body Mass Index (BMI) was obtained.

### Cultural adaptation of the Back Belief’s questionnaire

Using a five-step guideline proposed by Beaton and colleagues [19], the BBQ was translated into the Yoruba language. The protocol was done sequentially:

i. Forward translation of the items and response choice of the English version of the BBQ into Yoruba. Two professionally qualified translators bilingual in both the English and the Yoruba language and experienced in patient-related outcomes independently translated the English version of the BBQ into the Yoruba language. This stage involved two forward translations B1 and B2. One of the translators had information about the concepts being investigated in the questionnaire.
ii. Synthesis: A reconciliation meeting between the two translators and one researcher (CEM) produced a harmonized version (B-12).
iii. Back Translation: Two independent qualified translators translated the harmonized version (B-12) back into English (AT1 and AT2) to identify inconsistencies in the words and concepts of the harmonized version.
iv. Expert committee review: A panel comprising of two of the researchers and all the four translators met to discuss issues of cultural adaptation and linguistic equivalence with the original version of the BBQ. This expert committee review produced the pre-final version of the BBQ-Y.
v. Pre-testing: Thirty Yoruba speaking patients with LBP completed the pre-final version of the BBQ-Y. Important themes explored included the perception, comprehension, and interpretation of the translated items, various terminologies used, and formatting of the questionnaire.
vi. The participants’ interpretation of the items was then evaluated (during a cognitive debriefing interview) to see whether the adapted kept equivalence to the items of the English version. Reports at each stage covering the issues faced and how they were resolved were documented.

### Outcome measures

Back Beliefs Questionnaire - The BBQ, a specific self-reported questionnaire to explore beliefs and thoughts related to low back pain was developed by Symonds et al. [10]. The aim of the BBQ is to determine various inevitable consequences of LBP in a patient’s future among 14 determinants. The developers of the BBQ reported that the instrument had excellent internal consistency (Cronbach α: 0.7) and test-retest reliability (ICC: 0.87) [10]. The BBQ-Y was also used in this study. The Visual Analogue Scale (VAS) - This is arguably the most common and simple scale in pain research and practice [23]. The scale represents pain intensity dimension by a 10 cm plain line with two anchors points of “no pain” and “the worst pain I ever felt”. The patient marks on the 10 cm line, a point that best describes his or her pain level. The VAS is sensitive and reliable [23]. Odole and Akinpelu translated the VAS into the Yoruba language [24]. The study showed a significant moderate correlation between the VAS-Y and the original English version [r = 0.63(Cl 0.49–0.69); *p* < 0.05].

### Statistical analysis

Data were assessed for normality using visual (normal distribution curve and Q-Q plot) and statistical methods (Shapiro-Wilk’s test and Skeweness/Kurtosis scores). Descriptive statistics of mean, standard deviation, median and interquartile range were used to summarize data. Reliability of the BBQ-Y during test-retest phase was determined using Intra-Class Correlation (ICC 2, 1), and the Bland-Altman plot. The ICC values were rated as high (> 0.75), moderate (0.4–0.75) and low (< 0.4) agreements [25]. In addition, standard error of measurement (SEM) and minimal detectable change (MDC) were used to evaluate the reliability of the BBQ-Y. MDC refers to the amount of change in a score required to distinguish a true performance change from a change because of chance [26]. The MDC was calculated using the SEM (i.e. standard deviation of observed test scores for a true test score). The SEM and the MDC of the BBQ-Y were calculated using standardized methods.

The internal consistency of the BBQ-Y was determined using Cronbach’s alpha. Cronbach’s α if-item-deleted assessed the individual items’ contribution to internal consistency and redundancy. A Cronbach’s α of at least 0.70 is acceptable for outcome measures [27]. Using Spearman ranks correlation analysis (BBQ-Y data was not normally distributed) the convergent validity of the BBQ-Y with pain intensity using the VAS was carried out. Following a convergent hypothesis, the BBQ would be correlated with pain intensity. The authors determined the factor structure of the BBQ-Y using Exploratory Factor Analysis (principal component analysis) with Varimax rotation. Indices such as the Kaiser-Meyer-Olkin value (0.742), Bartlett’s test of sphericity (*X*^2^ = 313.97, *p* < 0.001) and the correlation matrix (preponderance of coefficients > 0.3) confirmed the appropriateness of the BBQ-Y data for PCA. To assess the acceptability of the BBQ-Y, the number and the proportion of the overall and for each item absence of responses were considered. Items were acceptable when the proportion of “no” responses was lower than 5%, and disputable if the proportion was higher than 10%. The ceiling and floor effects of the BBQ were established only if more than 15% of 119 participants scored the highest and lowest scores, respectively [28]. Alpha level was set at *p* < 0.05. Data analysis was carried out using IBM SPSS (Statistical Package for Social Sciences) for Windows version 23.0, Armonk, NY; IBM Corp..

## Results

Some adaptations were made to connote the meaning of some words in the original version. The word ‘belief’ was translated as ‘ìgbàgbọ́’ because that single word means both belief and faith in Yoruba. Also, ‘Back trouble’ was translated as ‘Ìṣòro tó rọ̀ mọ́ ẹ̀yìn’ (pain related to the back region) because of the direct translation ‘Ìṣòro ẹ̀yìn’ may give a negative connotation. In question 5, the subject of the sentence became the object on translation to make a meaningful sentence, ‘A bad back should be exercised’ became ‘A ni láti se eré ìdárayá pẹ̀lú ẹ̀yìn tí kò dára’.In Question 8, the sentence was not translated directly because direct translation may not communicate the meaning, so the sentence ‘Back trouble may mean you end up in a wheelchair’ was translated as ‘Ìṣòro tó rọ̀ mọ́ ẹ̀yìn lè túmọ̀ sí pé ori àga aláyíká ni o ti maa lo ìyókù ọjọ́ ayé rẹ’.In Question 13,‘Back trouble must be rested’ became ‘Ó di dandan kí a fún ẹ̀yìn tó ni ìṣòro ní ìsinmi’ on translation because direct translation could also not convey the meaning so the subject became the object on translation. 119 respondents (56.3% females) took part in the construct validity phase while 51 respondents (52.9%) agreed to take part retest phase of the reliability of the Yoruba version of the BBQ (BBQ-Y).

**Fig. 1:**
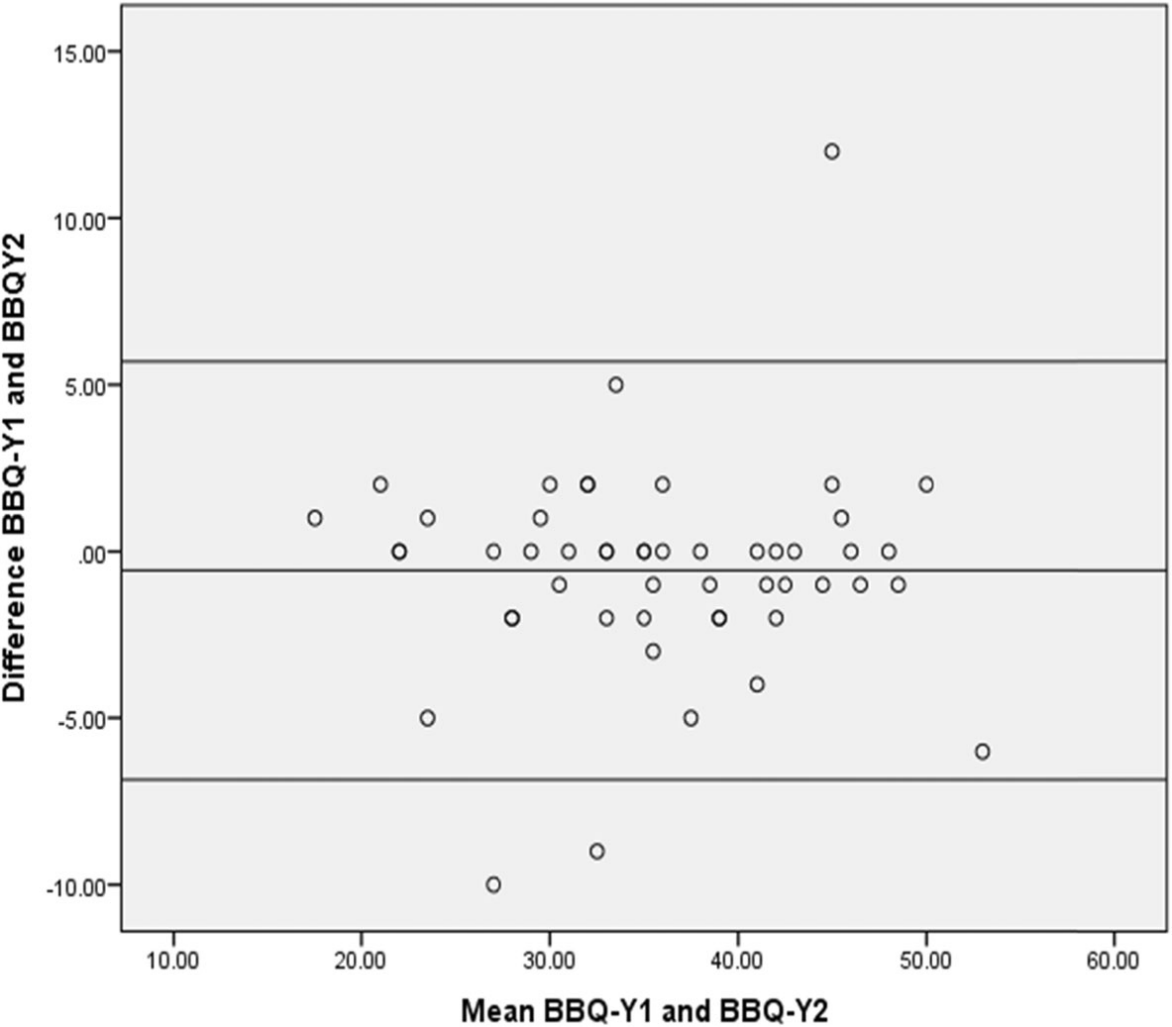
Bland–Altman plot for the test-retest of the Yoruba version of the Back Beliefs Questionnaire Mean difference

The mean age, weight, height, and BMI of the respondents were 56.8 ± 8.5 years, 69.0 ± 9.4Kg, 1.6 ± 0.1 m, and 25.9 ± 3.8 kg/m2 respectively. Respondents’ characteristics across the validity and reliability phases are presented in Table 1. The ICC of the global score was 0.89. Item by Item ICC values was significant for the BBQ-Y (*p* < 0.001) (Table 2). The Bland-Altman plot (Fig. 1) showed a mean difference of − 0.57 (5.70, − 6.84). The Cronbach’s alpha of the BBQ-Y was 0.71. Internal consistency score on the deletion of any item of the BBQ-Y shows that deletion of items 1 and 13 would increase the score to 0.72 and 0.71, respectively (Table 2). The SEM and minimum detectable change of the BBQ was 2.3 and 6.4 respectively. The convergent validity of the BBQ-Y was r = 0.273 at *p* = 0.001 based on the Spearman ranks correlation with pain intensity using the VAS-Y.

**Table 1:** Participants’ characteristics across the validity and reliability phases by gender

Variables	Validity				Reliability			
	Male Mean ± SD	Female Mean ± SD	t-value	*p*-value	Male Mean ± SD	Female Mean ± SD	t-value	*p*-value
Age (Years)	59.0 ± 8.3	55.1 ± 8.4	2.55	0.01**	62.5 ± 9.8	56.2 ± 8.1	2.51	0.02
Height (m)	1.7 ± 0.1	1.6 ± 0.1	4.38	0.01**	1.7 ± 0.1	1.6 ± 0.1	3.03	0.001
Weight (Kg)	69.4 ± 9.0	68.6 ± 9.7	0.50	0.62	70.0 ± 9.2	67.0 ± 8.2	1.25	0.22
BMI (Kg/m^2^)	25.1 ± 3.6	26.5 ± 3.9	−2.0	0.52	25.1 ± 3.5	25.7 ± 3.7	−0.62	0.54
	**Median (**IQR)	**Median (**IQR)	**U**	***p-value***				
VAS score (cm)	5.5 (3.0)	5.0 (2.0)	51,549.5	0.30				
BBQ-Y score	24.0 (8.8)	23.0 (9.0)	1580.	0.39				

*BMI* Body Mass Indexm, *VAS* Visual Analogue Scale, *SD* Standard Deviation, *BBQ-Y* Yoruba version of the Back Beliefs Questionnaire, *IQR* Interquartile Range;**-Significance at *p* < 0.05

**Table 2:** Internal consistency score if items of the BBQ-Y is deleted (*N* = 119; distractor items not included) and Item by Item Correlation between the Test-Retest of the Yoruba version of BBQ-Y (*N* = 51)

Item	Cronbach’s alpha if item deleted	Intra Class Correlation	95% Confidence Interval	
			Lower Bound	Upper Bound
1. There is no real treatment for back trouble	0.72	0.89	0.81	0.94
2. Back trouble will eventually stop you from working	0.69	0.95	0.91	0.97
3. Back troubles mean periods of pain for the rest of one’s life	0.68	0.91	0.84	0.95
4. Doctors cannot do anything for back troubles	a	0.92	0.85	0.95
5. A bad back should be exercised	a	0.94	0.89	0.97
6. Back trouble makes everything in life worse	0.65	0.95	0.92	0.97
7. Surgery is the most effective way to treat back trouble	a	0.91	0.83	0.95
8. Back trouble may mean you end up in a wheelchair	0.68	0.88	0.73	0.94
9. Alternate treatments are the answer for back troubles	a	0.83	0.70	0.90
10. Back trouble means a long period of time off work	0.67	0.97	0.94	0.98
11. Medication is the only way of relieving back trouble	a	0.91	0.87	0.92
12. Once you have back trouble, there is always a weakness	0.68	0.90	0.82	0.94
13. Back trouble must be rested	0.71	0.81	0.66	0.89
14. Later in life, back trouble gets progressively worse	0.68	0.97	0.95	0.98

*BBQ-Y* Yoruba version of the Back Beliefs Questionnaire; ^a^ – Distractor items not included during the internal consistency analysis

Results from a parallel analysis suggested keeping a two or three-factor solution. For the three factor structure, exploratory factor analysis showed that factors explained a total variance of 44.99% (Table 3). Except for items 1, 12 and 13, the first factor comprised the same items used in the BBQ’s scoring described by the developers. Only item 7 loaded along with the scoring items, while other distracter items loaded on the second or third factors (Table 3). Two factor solutions (factor one: items 1, 3, 4, 6, 7, 8, 10, 11 and factor 2: items 2, 9, 12, 13) explained 36.21% of total variance. However, items 5 and 14 did not load on any of the factors (Table 4). The BBQ-Y had no floor or ceiling effects, as none of the participants had either the lowest or highest scores.

**Table 3:** Factor loadings for the three-factor solution derived by principal axis extraction and Varimax rotation of the BBQ-Y

Item	Factor loading		
	Factor 1	Factor 2	Factor 3
1. There is no real treatment for back trouble		0.44	
2. Back trouble will eventually stop you from working	0.36		
3. Back troubles mean periods of pain for the rest of one’s life	0.67		
4. Doctors cannot do anything for back troubles		−0.62	
5. A bad back should be exercised		0.47	
6. Back trouble makes everything in life worse	0.72		
7. Surgery is the most effective way to treat back trouble	0.57		
8. Back trouble may mean you end up in a wheelchair	0.54		
9. Alternate treatments are the answer for back troubles			−0.71
10. Back trouble means a long period of time off work	0.63		
11. Medication is the only way of relieving back trouble		−0.36	**− 0.65**
12. Once you have back trouble, there is always a weakness			−0.78
13. Back trouble must be rested		0.40	
14. Later in life, back trouble gets progressively worse	**0.68**	0.34	
Percentage of total Variance (%)	25.92	10.29	8.78

*BBQ-Y* Yoruba version of the Back Beliefs Questionnaire; Significant loadings are mostly considered to be > 0.30. Bold indicates the highest factor loading for each item

**Table 4:** Factor loadings for the two-factor solution derived by principal axis extraction and Varimax rotation of the BBQ-Y

Item	Factor loading	
	Factor 1	Factor 2
1. There is no real treatment for back trouble	**0.46**	
2. Back trouble will eventually stop you from working		**0.45**
3. Back troubles mean periods of pain for the rest of one’s life	**0.58**	
4. Doctors cannot do anything for back troubles	**0.61**	
5. A bad back should be exercised		**0.36**
6. Back trouble makes everything in life worse	**0.70**	
7. Surgery is the most effective way to treat back trouble	**0.60**	
8. Back trouble may mean you end up in a wheelchair	**0.55**	
9. Alternate treatments are the answer for back troubles		**0.61**
10. Back trouble means a long period of time off work	**0.55**	0.33
11. Medication is the only way of relieving back trouble	**0.50**	0.36
12. Once you have back trouble, there is always a weakness	0.35	**0.58**
13. Back trouble must be rested		**0.69**
14. Later in life, back trouble gets progressively worse	**0.39**	**0.39**
Percentage of total Variance (%)	25.92	12.29

*BBQ-Y* Yoruba version of the Back Beliefs Questionnaire; only factors above 0.30 are shown. Bold indicates the highest factor loading for each item

## Discussion

This is the first study to culturally adapt the BBY-Y and determined its psychometric properties. The study provided the translation, cultural adaptation and psychometric properties of the Yoruba version of the BBQ among patients with chronic LBP, following the Guillemin criteria. The BBQ had good psychometric properties, comparable to other versions of the BBQ. The BBQ-Y showed excellent test-retest reliability (0.89). The findings of this study conform to the recommendation of an ICC of 0.75 or more, regarded as excellent in the literature [25]. The ICC coefficient of the BBQ-Y is higher than that reported in previous studies including the Modern Standard Arabic (0.80) [15], traditional Chinese (0.85) [29], original English (0.87) [10], the Simplified Chinese (0.88) [16] and the Arabic (0.88) [17] versions. Alongside the ICC, we explored the limits of agreement between test and retest data. ICC is commonly used to show proportion of the variability in the new method due to the ‘normal’ variability between individuals. One shortcoming of ICC is that it might indicate strong correlation between two measurements with minimal agreement. Bland-Altman accounts for this shortcoming by revealing both systematic and random errors during a test-retest analysis [30]. The results of this study on the limits of agreement indicates that the small measurement error was equally spread across the whole scale range.

The SEM and MDC of 2.3 and 6.4 obtained in this study suggest good clinical utility of the BBQ-Y. Changes in scores of two points and more on the BBQ have been reported to be clinically significant [31, 32]. However, a minimally clinically important difference (MCID) that takes into cognisance patients’ self-reported improvement and SEM has not been calculated for the BBQ in previous studies. Between-known groups mean is another alternative when an MCID is not obtainable [33]. A between-known groups mean difference of 20 points on BBQ scores among individuals off work because of LBP and those still working has been reported [10]. As such, in the absence of MCID, between-known groups mean difference could be used to interpret the observed limits of agreement. Therefore, the observed limits of agreement of the BBQ-Y are acceptable because they are smaller than the differences between “known groups”. Furthermore, MDC can serve as an indicator of true change in an individual. For instance, a difference in scores which exceeds that of the MDC upon two successive measurements can be viewed as a true change [34]. Thus, clinicians can use the MDC and SEM values of the BBQ-Y obtained in the current study to interpret the efficacy of interventions targeted at mal-adaptive back pain beliefs.

The BBQ-Y showed good internal consistency, as shown by the Cronbach’s coefficient of 0.7. Although slightly lower, the Cronbach’s coefficient of the BBQ-Y was comparable to those found in other translations [10, 15, 35, 36]. While our study focussed on persons with chronic LBP, others studies included patients with and without LBP [10, 16, 35, 36]. The results of this study further showed that removing items 1 (i.e. There is no real treatment for back trouble) and 13 (i.e. Back trouble must be rested) did not significantly change the homogeneity of the items within the scale. This is similar to the findings by Maki and colleagues [15] where BBQ still kept higher internal consistencies without including items 1 and 13. However, the authors in this present study, do canvas for the retention of the two items, as all nine items still contributed to the overall excellent internal consistency of the BBQ-Y.

Factor analysis of the BBQ-Y revealed a three-factor structure with 6 of the scoring items loading on one factor. The other 3 scoring items alongside the distractor items loaded on the remaining two factors. This is contrasting to the two-factor structure original version where all the scoring items loaded on one factor and the distractor items loading on the other factor. Most of the studies that conducted EFA on the BBQ-Y reported a three-factor structure [17, 35, 36]. Item 1 did not load along with the scoring items in the present study as well as the previous studies [17, 35, 36] that conducted an EFA on the BBQ. This item has been argued to have ambiguous meaning or measuring a different construct as the other items included in the final score of the BBQ [17, 35]. It is therefore likely that this item has a different connotation to the Yoruba speaking patient with LBP compared to the original validation population. While all other scoring items loaded into the same factor in the previous translations, items 12 and 13 did not for the BBQ-Y. Deleting Items 1 and 13, however, from the BBQ-Y will not significantly reduce the internal consistency of the instrument. However, to keep the original structure of the BBQ, items 1 and 13 may be retained. On the other hand, deleting item 12 would reduce the internal consistency of the BBQ-Y. It is therefore important that the item be retained in the total BBQ-Y score. The distractor item “Surgery is the most effective way to treat back trouble” loaded with the scoring items. Distractor items, including item 7, are often omitted from the final score of the BBQ irrespective of the factor loading, to reduce the time and cost of data collection [35]. Considering the two factor structure, the loading was less interpretable as both distractor and scoring items loaded in the two factors.

Chronic LBP beliefs and its interpretation are influenced by cultural upbringing, sociocultural environment, previous pain experiences and health literacy [37]. For instance, beliefs about LBP may be under-reported in the African settings due to cultural or religious reasons. Although, patient beliefs and other psychosocial factors influences LBP in comparable ways both in the developed and developing countries [38], culture can mediate how these beliefs and psychosocial factors are expressed. Thus, the difference in the factor structures of the different versions of the BBQ may be adduced to the differences in culture.

Based on difficulty and quality rating, the Yoruba version of the BBQ had a high rate of data completion with good quality data in the study population. The BBQ-Y achieved a response rate of 100% and did not have any ceiling or floor effect. We did not report non-responses to questionnaire items as such event was rare owing to the method of administration of the questionnaires, as the researchers hand-delivered the questionnaires and patiently waited to collect them. This method is still the preferred approach in many sub-Saharan African countries as the postal and electronic methods often fail due to technical and infrastructural challenges. However, we consider not having the details of non-response a shortcoming. The above findings suggest that the Yoruba version of the BBQ is an acceptable outcome measure for assessing back pain beliefs among the Yoruba-Speaking LBP patients. There was no significant structural alteration made to the BBQ-Y, other than required cultural-adaptation.

In summary, the moderate to excellent psychometric properties of the BBQ-Y lend credence to its usability and applicability in the clinic setting among patients with chronic LBP. The new tool may promote assessment of beliefs about chronic pain and also inform interventions to improve health outcomes of Yoruba speaking patients with chronic LBP. Anecdotal and empirical reports indicate that African patients with chronic pain have psychosocial problems, in this case, unhelpful beliefs, that have strong cultural undercurrents which in turn affect their health seeking behaviour [39, 40]. Early identification of patients with unhelpful beliefs about LBP is of utmost importance. This will help to focus interventions on those maladaptive beliefs rather than approaching the LBP problem from a biomedical perspective. Interventions incorporating the bio-psychosocial framework are recommended as the most effective approach in managing LBP [41]. Unlike the biomedical approach, bio-psychosocial interventions not only treat impaired anatomical structures but may also address psychological, socioeconomic, ecological and cultural factors that may impact on the onset and persistence of LBP.; hence, the importance of such translation as the BBQ-Y.

### Limitations and methodological problems

Limitations of the study include the cross-sectional nature of the study, which did not allow for the examination of other aspects of validity, including sensitivity and responsiveness. Second, the authors conducted the study within the clinical settings, thus may not totally represent individuals with LBP in Nigeria. Also, we did not investigate the construct validity of the BBQ-Y with other measures commonly used in the literature, including the fear-avoidance beliefs questionnaire and the Tampa scale of kinesiophobia. Furthermore, we did not conduct confirmatory factor analysis in this study, as a large sample size will be required for it.

### Implications for future research

Future research on the BBQ-Y using larger and more diverse sample is required. In addition, further psychometric testing on convergent validity and factor structure are recommended for future studies.

## Conclusion

The Yoruba version of the BBQ is a valid and reliable instrument for LBP. The psychometric properties of the BBQ-Y are consistent with the original English version and other translations. This study has provided a valid tool for assessment of back beliefs about LBP among Yoruba patients.

## Data Availability

The supporting data for this study is available as supplementary material.

## Abbreviations

BBQ: Back beliefs questionnaire
BBQ-Y: Translated yoruba BBQ
ICC: Intra-class correlation
LBP: Low-back pain
VAS: Visual analogue scale
VAS-Y: Yoruba version of the visual analogue scale
SEM: Standard error of measurement
MDC: Minimal detectable change
SPSS: Statistical package for social sciences
BMI: Body mass index
MCID: Minimally clinically important difference
